# Mining Bodily Cues to Deception

**DOI:** 10.1007/s10919-023-00450-9

**Published:** 2024-01-16

**Authors:** Ronald Poppe, Sophie van der Zee, Paul J. Taylor, Ross J. Anderson, Remco C. Veltkamp

**Affiliations:** 1 Information and Computing Sciences, Utrecht University, Utrecht, The Netherlands; 2 Applied Economics, Erasmus School of Economics, Erasmus University Rotterdam, Rotterdam, The Netherlands; 3 Erasmus School of Law, Erasmus University Rotterdam, Rotterdam, The Netherlands; 4 Psychology, Lancaster University, Lancaster, UK; 5 Psychology, University of Twente, Enschede, The Netherlands; 6 Computer Laboratory, University of Cambridge, Cambridge, UK; 7 Security Engineering, School of Informatics Institute for Computing Systems Architecture, University of Edinburgh, Edinburgh, UK

**Keywords:** Body motion, Motion capture, Movement analysis, Deception, Data mining

## Abstract

A significant body of research has investigated potential correlates of deception and bodily behavior. The vast majority of these studies consider discrete, subjectively coded bodily movements such as specific hand or head gestures. Such studies fail to consider quantitative aspects of body movement such as the precise movement direction, magnitude and timing. In this paper, we employ an innovative data mining approach to systematically study bodily correlates of deception. We re-analyze motion capture data from a previously published deception study, and experiment with different data coding options. We report how deception detection rates are affected by variables such as body part, the coding of the pose and movement, the length of the observation, and the amount of measurement noise. Our results demonstrate the feasibility of a data mining approach, with detection rates above 65%, significantly outperforming human judgement (52.80%). Owing to the systematic analysis, our analyses allow for an understanding of the importance of various coding factor. Moreover, we can reconcile seemingly discrepant findings in previous research. Our approach highlights the merits of data-driven research to support the validation and development of deception theory.

## Introduction

There is much interest in estimating whether a subject is telling the truth, for example in police interviews or in border screening. Over the past decades, a significant body of research on deception has emerged (Denault et al., 2022). One prominent topic concerns the verbal and nonverbal cues that can distinguish a truth-teller from a liar (Vrij, 2008). Most of the research into relevant behavior cues has followed the approach of empirically testing hypotheses derived from psychological models of communication and personality. Increasingly, there is criticism on this approach, pointing at a lack of generalization across settings as a result of inflated statistical reporting (Levine, 2018b). Moreover, the manual coding of cues to deception is under scrutiny, as are the reported effect sizes of these cues (Luke, 2019). As a result, the practice and merits of deception research as a whole is questioned (Brennan & Magnussen, 2020).

At the same time, deception detection has gained interest from the technical sciences, with the aim of increasing detection rates. This applied research does not rely on theories of deception, but rather considers patterns in the data directly. Data mining systems are built with the philosophy of collecting as much data as possible, extracting many signals from it, and then using statistical machine learning techniques to work out which combinations of signals have discriminatory power (Baltrušaitis et al., 2019). In recent years, significant progress has been made with this approach (Avola et al., 2020; Ding et al., 2019; Wu et al., 2018). Yet, data mining research is often being criticized for its lack of interpretability, overfitting to specific populations or settings, and the potential risk of biased judgements. Overfitting refers to the issue that a model does not generalize beyond the data that was used to develop it. In the context of deception research using machine learning approaches, similar observations are made (Constâncio et al., 2023). Especially when employing deception detection in practice, these factors severely limit the acceptance of such systems (Sánchez-Monedero & Dencik, 2022).

Theoretical and applied research have different aims but they commonly focus on discovering informative behavioral cues that are linked to either being honest, or being deceptive. Owing to this shared focus, a recent view that data analysis research can aid in the understanding of behavior (Yarkoni & Westfall, 2017) is gaining traction. This paper supports this notion; we take a data mining approach to understand the behavioral factors that influence the detection of deception. The aim of this paper is explicitly not to achieve the best deception detection performance, but to assess the viability of a data-driven approach to aid in theory development and validation. To this end, we systematically vary various measurement options and observe their effect on the overall classification rate. We focus on body pose and body movement as possible indicators of deception, but our approach could easily extend to other modalities such as facial and verbal expressions. In the remainder of this section, we review the current challenges in deception detection research and the potential for automated methods to increase our understanding of the problem.

### Challenges in Deception Detection Research

Being deceptive is generally assumed to be more cognitively demanding (Vrij et al., 2010), and might lead to higher levels of arousal (de Turck & Miller, 1985). These factors might affect bodily, facial and verbal behavior, thereby “leaking” cues to deception (Ekman & Friesen, 1969). In line with other modalities, the literature on bodily cues to deception is characterized by inconsistent and often contradictory findings (Levine, 2018b). Meta-analyses (DePaulo et al., 2003; Sporer & Schwandt, 2007) have revealed that very few cues are correlated consistently across studies. This might be partly caused by the different processes associated with lying. These include emotional responses, increased cognitive load and attempted behavioral control, each of which can lead to different types of behavior (Vrij, 2008). For example, when people are aware that lying-induced arousal can cause an increase in movements seen as indicative of lying, the behavioral control theory predicts that liars will try to control their movements to appear honest. This might lead to rigid and unnatural movement (Buller & Burgoon, 1996). For example, leg movements have been found to both decrease (Levine et al., 2005) and increase (Davis et al., 2005; Van der Zee et al., 2019) when lying.

The different cognitive and affective processes thus may elicit contradicting cues, potentially simultaneously (Vrij et al., 2019). Consequently, differences in behavior between liars and truth-tellers are expected to be subtle, perhaps too subtle to be reliably perceived by human observers. This might be another cause for the inconsistent findings in deception detection research (Bond & DePaulo, 2006).

Partly due to their limited perception skills, humans perform poorly at deception detection, achieving detection rates only a couple of percent better than tossing a coin (Vrij, 2008). In addition, humans typically have a judgement bias (Meissner & Kassin, 2002). For example, police officers suffer from a guilt bias, whereas the general public usually tends to hold a truth bias. Recently, advances in interviewing techniques have slightly increased human detection rates (Deeb et al., 2022), by asking questions in reverse-order (Blandón-Gitlin et al., 2015; Vrij et al., 2008) or asking unanticipated questions (Vrij & Granhag, 2012) to increase cognitive load. Training also has been shown to moderately increase detection rates (Hauch et al., 2016). Still, comparisons between human and automated detection of deception are in favor of the machines, despite efforts in combining their relative strengths (Kleinberg & Verschuere, 2021).

We argue that the automated analysis of bodily behavior can lead to improvements in terms of deception detection in practice, with increased detection rates and a reduction in subjective bias. More importantly, we see opportunities to improve our understanding of deceptive behavior by relying on detailed, objective measurements instead of human perception.

### Automated Analysis of Body Movement

In addition to the use in practical applications such as border screening and interviewing, the automated analysis of body movement can be used to discover novel cues, or to empirically validate or develop deception theories, in line with Yarkoni and Westfall (2017). Relying on automated measurement circumvents the issue of perception bias, and allows for a more fine-grained analysis: instead of focusing on a limited set of discrete behaviors, many cues at various levels can be taken into account, including the precise direction, magnitude and timing of movements (Poppe et al., 2014). Thus more subtle cues can be evaluated.

Similar to facial expressions, bodily behavior can be measured unobtrusively. The automated measurement and analysis of facial and bodily cues from camera footage has seen a lot of progress in recent years (Baltrušaitis et al., 2019). This is particularly true for facial expression research, which can rely on a commonly used representation system: the facial action coding system (FACS, Ekman & Friesen, 1978). Both human coders and machines can be trained to provide the numerical representations in the FACS standard. This facilitates the adoption of knowledge of the behavior sciences for automated detection. Vice versa, automated analysis can be more easily used for theory verification and development. Unfortunately, for the analysis of body movement, there is no such commonly agreed on quantitative representation system (Poppe, 2017). Bodily expressions are arguably more complex to represent than facial expressions because of the larger number of degrees of freedom which gives a wide range of possible body poses. There have been some recent efforts in developing coding schemes (Dael et al., 2012; Poppe et al., 2014) but these are not commonly used and are not suitable for both human and machine coders.

A second complicating factor for the automated measurement of bodily behavior is the lack of accurate ways to unobtrusively measure body pose and movement (Chen et al., 2020). Impressive advances in video-based analysis have been made in recent years. In addition, the availability of novel sensor technology such as depth cameras has resulted in a performance increase. Still, the robustness and accuracy is far from the gold standard of motion capture technology. This is largely due to partial occlusions of the body that cause some body parts to be invisible from the perspective of the camera.

When applied to deception detection, these limitations have forced researchers to focus on constrained recording conditions (Elkins et al., 2014). For example, Lu et al. (2005) detected the hands of a subject using skin color and blob analysis. They subsequently analyzed the location and the trajectories of the hands, relative to the face and body. With such analyses, gross differences in hand movements, including face touches, can be considered. This approach has been refined by Jensen et al. (2008) to include various geometric properties to describe to relative position and movement of the hands and head. Movements of children are analyzed by a simple frame-differencing method in Serras Pereira et al. (2016). Such methods are prone to issues of robustness because factors such as clothing and distance to the camera affect the measurements.

To circumvent the limitations of video recordings, both Duran et al. (2013) and Van der Zee et al. (2019) have analyzed motion capture data. Interestingly, they found different effects of lying on observed body movement. When observing body movement over small time windows, Duran et al. (2013) found no difference in the amount of motion but did observe a change in the stability and complexity of the motion patterns. In contrast, Van der Zee et al. (2019) observed an increase in body movement, replicated in each limb, when lying. In their analysis, aggregate measures of body movement over 2.5 min were used, essentially ignoring brief motion patterns.

Such seemingly discrepant results are likely to be caused by the deception setting or the measurement of the bodily behavior. To address the second source of variation, this paper focuses on understanding the effect of coding variables such as body part, the coding of the pose and movement, the length of the observation, and the amount of measurement noise. We mine motion capture data for bodily cues to deception. Instead of employing complex mechanisms to exploit feature correlations, we explicitly focus on gaining insight in the type of signals that can be used in a practical application, but also to verify or develop deception theories.

The paper is organized as follows. We first describe our methodology, with data collection and annotated. We then summarize our main results. Finally, we discuss our findings and implications for deception research.

## Methodology

We use the data described in Van der Zee et al. (2019). We briefly outline the setup of their experiment, and then proceed with the detailed description of our coding of the recorded body movement. Then, we discuss our classification procedure.

### Data Collection

#### Participants

In total, 180 students and employees, divided into 90 pairs, took part in the experiment. People with either White British or South Asian cultural background were explicitly included in the study. In the present study, we only consider the *n* = *90* interviewees, of which 60 were born in South Asia while the remaining 30 were British, according their own reports. The average age was 22.37 years (range 18–39). A total of 54 participants (60.0%) self-identified as female. Van der Zee et al. (2019) found no statistically significant effect of the subjects’ cultural background on the amount of whole body movement. Therefore, and to ensure a sufficiently large sample size, we refrain from examining the effect of the cultural background and gender. The experiment was approved by the Lancaster University Research Ethics Committee.

#### Procedure

Interviewees were randomly assigned to a *truth* or *lie* condition. In both cases, interviewees performed two tasks prior to the interview. In the truth condition, they played a computer game *Never End* and delivered a wallet to the lost-and-found. Never End is a 2D platform game in which the player must escape from a maze. They were told that the wallet was from a previous participant and that the experimenter would send an email while the participant would deliver it. In the lie condition, they only looked at a description of the game, and were instructed to take a 5 pound note from the wallet and hide the note on their body. The interviewer had to be convinced that the wallet was handed in at the lost-and-found, and the game was actually played.

After being instructed, giving consent and providing demographic information, pairs of two subjects (the *interviewer* and *interviewee*) were seated facing each other. The interviewer asked the interviewee a number of questions in a fixed order, by reading them out loud from paper. For the *Game* session, these questions were in reversed chronological order, adding to the difficulty of the task (Vrij et al., 2008). In the *Wallet* session, questions were asked in normal order but the stakes were arguably higher because the lie involved taking money.

During the interview, the vast majority of the time the interviewee was answering the questions. In both the truth and lie conditions, interviewees were tasked with convincing the interviewer that they were telling the truth. In the lie condition, all answers had to be deceptive. Sessions lasted about 2.5 min, and were then stopped.

### Data Coding

In this study, we only use the recorded body movement for analysis. The body movements of interviewers and interviewees were recorded with Xsens MVN motion capture systems. These employ inertial sensors placed in straps around the body to measure the 3D position of 23 joints in the body at a rate of 60 measurements per second. No post-processing has been applied.

**Fig. 1:**
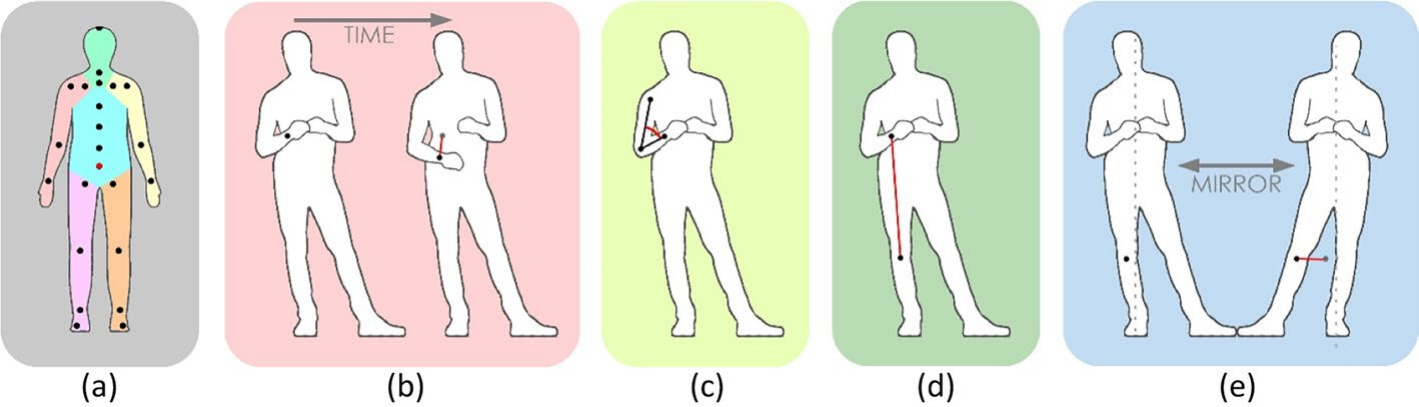
From left to right: **a** Location of the 23 joints. Root joint in red. Body parts are indicated with different colors. **b**–**e** Schematic visualization of the four feature types: movement, joint angle, joint distance, and symmetry

Figure 1a shows the locations of these joints. We use only the data of the interviewee. There might be meaningful patterns in the coordination of the behavior of both interactants (Dunbar et al., 2014; Duran & Fusaroli, 2017; Van der Zee et al., 2021), but we leave this for future work. Body movements were continuously recorded over the 2.5 min interview. No distinction was made who spoke.

#### Space Dimension

In line with Poppe et al. (2014), we normalized body posture data for global position by expressing joint positions relative to the root (i.e., pelvis). We also scaled all body parts to average lengths, to overcome differences in body dimensions between subjects. These transformations can be made without any knowledge of the subject. The resulting representation is a 66-dimensional coordinate system (22 3D joint positions). From this representation, we calculated a number of features grouped in four different *feature types*. There is a large body of research (e.g., Castellano et al. (2007); Kleinsmith and Bianchi-Berthouze (2013)) that has focused on the relation between body motion and affective or cognitive states. Our approach aligns with these works but our selection of features is by no means novel (see, e.g., Jensen et al., 2008), nor complete. Our aim is to use a representative set that covers different pose and motion qualities, across the body. A schematic overview of the features employed in this paper appears in Fig. 1b–e. We summarize the four feature types:

- Movement: We focus on the movement of individual joints by calculating the Euclidian distance between the joint position in two subsequent measurements. Additionally, we calculate the total amount of movement for the body parts left/right leg, left/right arm, torso and head, and for the upper and full body. The body parts are visualized with different colors in Fig. 1a. The upper body contains both arms, the torso and the head. Full body contains all body parts. The total number of features of the movement type is 30.
- Joint angle: Body movement occurs at the joints. Each joint has between one and three degrees of freedom, determined by the number of axes around which the joint can revolve. We do not regard these physical degrees of freedom, but rather calculate the smallest angle directly by only considering the plane in which the two neighboring segments of a joint reside. We then calculate the angle between the vectors of the two neighboring segments. For example, for the left elbow we consider the vector shoulder-elbow and the vector elbow-wrist. The joint angles that we consider are those of the neck, shoulders, elbows, hips, and knees. We also include the mean of these 9 angles, which brings the number of joint angle features to 10.
- Joint distance: The relative position of two body parts might be informative. For example, face touches have been attributed to deception (Ekman & Friesen, 1969). Additionally, these features can be used to distinguish between compact and more elongated postures. We calculate the Euclidian distance between pairs of joints: head-left/right elbow, left hand-right hand, left hand-right elbow, right hand-left elbow, left hand-left knee, right hand-right knee, left knee-right knee, left ankle-right ankle, and pelvis-right/left ankle. Including the average of these distances, we obtain 12 features.
- Symmetry: We left–right mirrored the joint positions in the plane through the root, perpendicular to the hips. We then compared the mirrored positions of all joints to the unmirrored positions of their left/right counterpart. We use the distance between each pair as a feature. Given that these are equal for left–right counterparts, we only calculated the features for the left limbs. We finally also calculated the average of these distances. This mean it is a measure for the symmetry of the whole body pose. In sum, this amounts to 15 symmetry features.

The total number of features that we extract is 67. Although these features are arbitrarily chosen, they cover the whole body, include both local and global descriptions, and carry a broad range of information.

#### Time Dimension

Xsens motion capture suits record data at a rate of 60 measurements per second. When using vision-based motion analysis, such high frequencies are not common (Chen et al., 2020). To ensure that the findings of our study can be more conveniently replicated with recent technology to record body pose from video material, we re-sampled our data down to 5 frames per second. Given that we aggregate the measurements over windows, the effect on the performance is minimal.

Not much is known about the time scale over which deception should be observed. Ideally, we would like to consider smaller windows as they would reduce the time needed to make a decision regarding the truthfulness of a subject's account. This would have great practical value. We therefore include the length of our observation window as a parameter of investigation in our experiments. Smaller windows allow for good representation and identification of brief, salient movements such as a face touch or a posture shift. But they might often be devoid of discriminative movement and thus uninformative. They may also fail to capture significant longer-term behavior. For larger windows, the opposite is true. To be able to compare our findings to those reported in the literature, one window setting considers the entire session duration of approximately 2.5 min; we also use increasingly smaller *window lengths* of 1 min, 30 s, 10 s, 5 s, and 1 s. For each window, we calculate the mean, minimum, maximum, range, and standard deviation of the feature values, which we will call *window types*. The windows are non-overlapping and the total dimensionality of the feature vector for each window, independent on the window size, is therefore 335 (67 × 5).

### Classification Procedure

Our aim is to discover individual cues that discriminate between truthful and deceptive accounts. To this end, we train classifiers for each feature individually on a training set, and subsequently evaluate the classifiers on test data. The data in the training and test sets are disjoint, which allows for the analysis of the generalization of the learned classifiers to unseen data, typically from other subjects. We use a leave-one-out cross-validation (LOOCV) approach, with the data of one pair (i.e., two sessions) in each fold. Specifically, we train on the data of *n* = *89* pairs and test on the remaining pair. We do this for all pairs and present results as the average detection rate over all 90 test folds.

Our classifier is the Gaussian Naive Bayes Classifier (Duda et al., 2000). It models the values of each class as a normal distribution. For each class *c* (truth or lie) and each feature *i* (*1* ≤ *i* ≤ *335*), we determine the mean value (${\mu }_{{c}_{i}}$) and standard deviation (${\sigma }_{{c}_{i}}$) of the feature on all training samples. Given a feature value *x*_*i*_ in the test set, we can determine the most likely class ${\widehat{c}}_{i}$:

$$\hat{c}_{i} = {\text{argmax}}_{c} \frac{1}{{\sigma_{{c_{i} }} \sqrt {2\pi } }}e^{{ - \frac{{\left( {x_{i} - \mu_{{c_{i} }} } \right)^{2} }}{{2\sigma_{{c_{i} }}^{2} }}}}$$

We assume equal prior probabilities for the two classes. This is common in lab settings, to which we compare our work. We discuss the consequence of this choice later.

With per-feature classification, we can classify our test data based on a single feature. This allows us to look at the predictive quality of an individual feature. Features with higher correct classification rates can be considered more promising features for deception detection. Additionally, we consider all features together using two different measures. First, we take the majority vote over the binary class estimates: the class which has been estimated by the majority of the per-feature classifiers is the guessed class. Second, we take the majority vote but only over the features whose distributions are statistically dissimilar with a probability of at least 95 and 99%, respectively. We will refer to these feature sets as *all, stat-95* and *stat-99*, respectively. While we consider multiple features jointly, we do not consider correlations between them as one would do with a typical machine learning approach.

## Results

In this section, we describe our main results and those of various computational experiments regarding the influence of window length, feature and window type, and noise.

We consider three sets of training and test data: (1) from both tasks together, (2) only from the Game sessions, and (3) only from the Wallet sessions. We evaluate all combinations of training and test sets to gain insight in the potentially different nature of the three sets. We use one feature vector per session, corresponding to a window length of approximately 2.5 min. As such, we use all available data. Classification results for *all*, *stat-95*, and *stat-99* appear in Table 1. Overall classification performance is 60.00% when training and testing on both sets on all features, and improves with another 5.00–5.56 percent point to 65.00–65.56% when only statistically significant features at the 5 and 1% are considered, respectively. The *all* set always contains all 335 features, while the average number of features over all tests with one subject left out in *stat-95* and *stat-99* is 136.96 (range 127–153) and 94.58 (range 85–110), respectively. In the remainder of this paper, we will focus on the *stat-95* features obtained when training on both deception tasks.

**Table 1:** Classification rates (in %) of different training and test sets, obtained using all/stat-95/stat-99 features, using windows of 2.5 min

	Trained on		
Tested on	Both	Game	Wallet
Both	60.00%/65.00%/65.56%	58.89%/61.67%/65.56%	62.22%/66.11%/64.44%
Game	64.44%/67.78%/68.89%	62.22%/64.44%/70.00%	64.44%/68.89%/64.44%
Wallet	55.56%/62.22%/62.22%	55.56%/58.89%/61.11%	60.00%/63.33%/64.44%

### Feature Type

Our feature sets consists of four feature types: movement, joint angle, joint distance, and symmetry features. In Table 2, we summarize the number of selected features in the *stat-95* set, as well as the average classification rates when only features of the selected type are used. We report the performance when testing on both sessions, as well as for each of the two sessions individually.

**Table 2:** Percentage of selected stat-95 features when training on both session types and average classification rate per feature type on both session types, Game session only and Wallet session only, using windows of 2.5 min

Feature type	Classification rate			
	Selected (%)	Both (%)	Game (%)	Wallet (%)
Movement	35.50	58.39	60.71	56.06
Joint angle	22.78	57.12	58.73	55.51
Joint distance	47.52	58.16	59.74	56.59
Symmetry	58.40	56.41	56.27	56.54

### Window Length

**Fig. 2:**
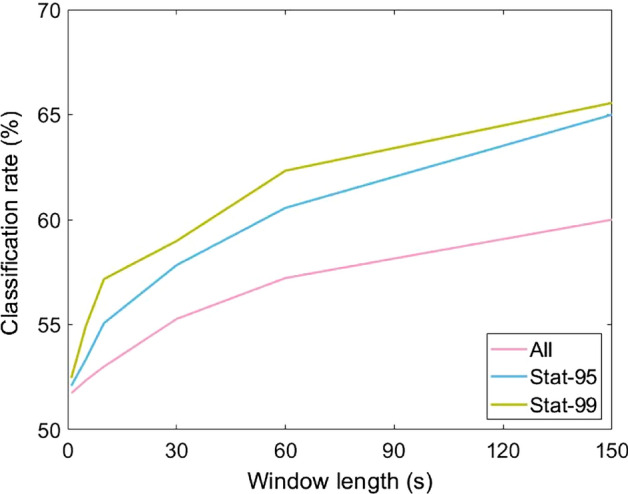
Classification scores in percentages for different window sizes (in seconds), obtained using all, stat-95 and stat-99 features

We evaluate six window lengths: 2.5 min, 1 min, 30 s, 10 s, 5 s, and 1 s. Classification rates for *all*, *stat-95*, and *stat-99* are summarized in Fig. 2.

### Window Type

Each feature was evaluated per window, for which we used five window types: mean, maximum, minimum, range, and standard deviation. Results are summarized in Table 3, for both session types together and each individually.

**Table 3:** Percentage of selected stat-95 features when training on both session types and average classification rate per window type on both session types, Game sessions only and Wallet sessions only, using windows of 2.5 min

Window type	Classification rate			
	Selected (%)	Both (%)	Game (%)	Wallet (%)
Mean	53.17	57.80	61.04	54.55
Maximum	43.90	58.42	57.73	59.12
Minimum	26.78	52.69	53.68	51.70
Range	37.94	59.38	59.53	59.22
Standard deviation	42.62	58.02	60.26	55.76

### Amount of Noise

**Fig. 3:**
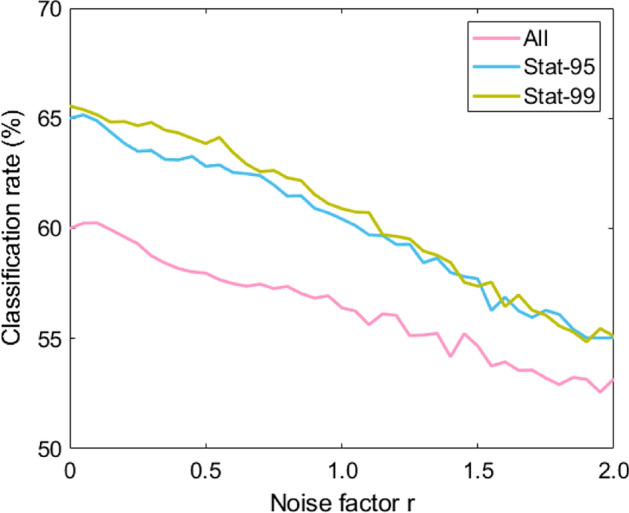
Classification scores in percentages for added noise with different factors *r*, obtained using all, stat-95, and stat-99 features. Scores are averaged over 100 repetitions

To test the classification performance in the presence of measurement noise, for example due to inaccurate measurements, we add noise on the motion captured test data. To each feature, we add Gaussian noise with a zero mean and a standard deviation *r* times the standard deviation of the feature in the training data (${\sigma }_{{c}_{i}}$). Adding Gaussian noise is somewhat artificial as noise is typically correlated in space and time, but it shows the robustness of the classification to inaccurate measurements. Results appear in Fig. 3.

## Discussion

We first discuss the effect of data coding choices on the deception detection performance. Then, we reflect on the merits and limitations of our data mining approach for deception detection.

### Effect of Data Coding

The classification performance of 65.00% for *stat-95* is statistically significantly higher than a naive baseline of 50% (*z* = 8.05, *p* < 0.001, 95% CI [0.46–0.54]). This performance demonstrates that predictive features can be mined from data. In Van der Zee et al. (2019), the interviewers also estimated the veracity of the interviewees. Their judgements were correct in 52.80% of the sessions. With 65.00%, the classification performance is significantly better than that of humans (*z* = 6.56, *p* < 0.001, 95% CI [0.49–0.56]). The superior performance of our automated approach seems to suggest that data can inform us of the presence or absence of cues that are related to deception.

The scores for the Game task are generally higher than those of the Wallet sessions (for *stat-95* 67.78 and 62.22%, respectively). This can be due to the more difficult nature of having to deceptively answer questions in reverse order (Vrij et al., 2008). This difficulty may have magnified the changes in behavior.

Overall, the best results are obtained when training on the Wallet sessions. When more training data is available, better classification rates are usually obtained. However, the additional availability of the Game sessions does not improve the results. Rather, the Game sessions appear to somewhat negatively affect the learning of the classifiers. This might be due to the more pronounced nature of the behavior in these sessions. The differences between truthful and deceptive accounts apparently do not generalize to other settings, specifically the Wallet sessions. When using fewer, statistically more significantly different features, classification rates typically increase. A notable exception is when the models are trained on the Wallet sessions. For *stat-99*, the classification rate is 64.44%, irrespective on the test sessions. This, again, seems to suggest that the patterns observed during the Wallet sessions generalize better, especially if these patterns already differ strongly significantly between truthful and deceptive accounts.

Table 4 and 5 show the confusion matrices of the classifier when trained on both tasks and tested on the Game and Wallet data separately. In both sessions, there is a truth bias. In the Game and Wallet sessions, respectively, 62.22 and 67.78% of the classifications are truthful. This leads to high recall rates for truthful accounts (80.00%), but markedly lower recall for deceptive ones, at 55.56 and 44.44% for the Game and Wallet sessions, respectively. We expect that this truth bias is due to the more varied nature of deceptive accounts. A systematic bias towards truthful accounts reduces the risk of false accusations, but at the cost of lower deception detection ability.

**Table 4:** Confusion matrix for Game sessions, trained on stat-95 features using windows of 2.5 min

	Actual		
Guessed	Truth (%)	Lie (%)	Total (%)
Truth	40.00	22.22	62.22
Lie	10.00	27.78	37.78
Total	50.00	50.00	100.00

**Table 5:** Confusion matrix for Wallet sessions, trained on stat-95 features using windows of 2.5 min

	Actual		
Guessed	Truth (%)	Lie (%)	Total (%)
Truth	40.00	40.00	67.78
Lie	10.00	10.00	32.22
Total	50.00	50.00	100.00

#### Influence of Window Length

Classification scores increase with window length: the additional information that is accumulated over time is beneficial for decision performance. Decisions about a subject's veracity become more reliable when the subject's behavior is observed over longer periods of time. For windows of a single second, the performance is barely above chance level. Performance increases with windows size, although with diminishing returns; an upper bound to the performance is to be expected.

The fact that there is more training data available for smaller windows does not help in the classification. Between the smallest (1 s) and the largest (2.5 min) windows, there is a factor 150 more training samples available. We hypothesize that many of these windows are uninformative which would reduce the efficacy of the classifier. This is especially true for generative classifiers such as our employed Gaussian Naive Bayes Classifier (Zhou et al., 2004). The situation is worsened because smaller differences between truthful and deceptive accounts will be statistically significant due to the larger number of available windows.

These results demonstrate the challenge in mining specific body cues. Clearly, aggregate information such as the average amount of body movement (Van der Zee et al., 2019) can be easily identified as a discriminative cue, while the discovery of briefer body motions such as those reviewed in DePaulo et al. (2003) is complicated by the variance in observed behavior over time.

Compared to *stat-95* scores, approximately twice the window size is needed to achieve similar results using *all* features. For smaller windows, a similar trend can be observed between *stat-99* and *stat-95*. As the number of features decreases from *all* to *stat-95* to *stat-99*, it appears that fewer features is beneficial to the classification. To test this hypothesis, we systematically varied the number of selected features from 1 to 200. Features were sorted on the significance level of the difference between the truth and lie feature distributions.

**Fig. 4:**
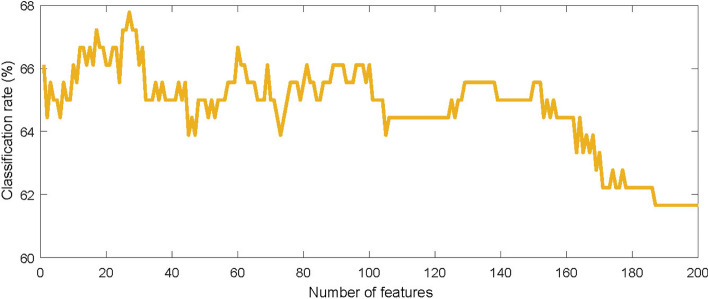
Classification scores in percentages for different numbers of features in decreasing order of statistical difference, obtained with a window size of 2.5 min

Figure 4 shows the classification rate as a function of the number of features used. Judging from Fig. 4, the optimal number seems to be around 25, or 7.5% of all features. Including more features decreases the classification performance as these features are less discriminating, and might cause the classifiers to overfit on the training data. A stricter selection on the level of significance of the difference between the distributions of truthful and deceptive samples is therefore recommended.

#### Influence of Feature Type

When looking at the different feature types, joint angle features have the lowest probability of being selected, whereas the majority of the symmetry features are selected in *stat-95*, see Table 2. Yet, differences in classification rate between these feature types are small and we found no large differences between the two session types. All types appear to contribute to the classification. Given that individual types all score below the combined score of 65.00%, they provide partly complementary information.

**Fig. 5:**
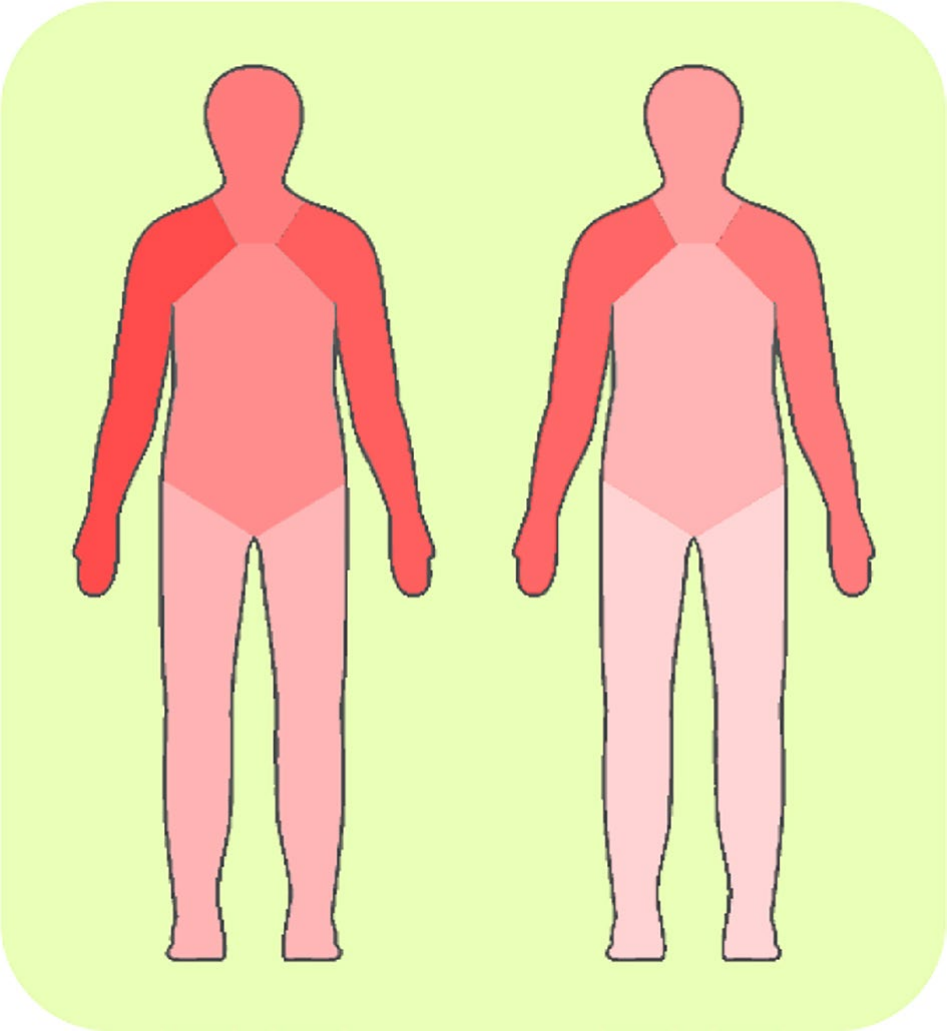
Visual representation of the percentage of features selected in stat-95 and stat-99. Darker colors correspond to higher percentages

The pool of features we evaluated covers all parts of the body. We analyze whether some body parts are more informative than others in the detection of deception. To this end, we indicate for each feature which body parts it considers. For example, the distance between the left hand and the right knee considers both the left arm and the right leg. Averages over all joints or distances take into account all body parts. Given that we thus link features to body parts, we can analyze how often these features contribute to the classification. We calculate, for each body part, the percentage of features linked to it that occur in *stat-95* and *stat-99*. Results are summarized in Table 6 and are visually represented in Fig. 5.

**Table 6:** Percentage of selected stat-95 features when training on both session types and average classification rate per body part on both session types, Game sessions only and Wallet sessions only, using windows of 2.5 min

Body part	Classification rate			
	Selected (%)	Both (%)	Game (%)	Wallet (%)
Left arm	70.02	60.18	61.19	59.17
Right arm	62.83	58.56	60.54	56.59
Left leg	29.32	56.96	58.00	55.92
Right leg	28.64	56.84	58.35	55.33
Head	51.36	58.21	59.53	56.88
Torso	44.79	58.59	58.79	58.40

There are large differences between body parts in the percentage of features that are selected. For *stat-95*, approximately 70% of the features in the left arm are selected, whereas a mere 30% of the leg features is found statistically different between the truth and lie conditions. The upper body plays a more prominent role in distinguishing truthful and deceptive accounts. This pattern is more visible in *stat-99*, with lower ratios of selected features. However, the selection of a feature is not indicative of its contribution to the classification performance. To understand this relation, we present the classification rates for each body part in Table 6. Differences between body parts in terms of classification rates are negligible, for both *stat-95* and *stat-99*. Also, there is no clear difference between the two session types, considering that the overall classification performance for the Wallet sessions is somewhat lower. When using features from all body parts, the classification rate is 65.00%. None of the body parts alone achieves comparable rates so different body parts are partly complementary in terms of the contribution to the classification.

Some features take into account a single body part whereas others use the positions of joints in two or more parts. We analyzed whether the extent of the feature, expressed in the number of body parts it takes into account, is of influence to the classification performance. See Table 7 for a breakdown of the results. The probability that a feature is selected increases with the number of body parts involved. This can be explained as the variance of a single feature is probably larger than the average of a number of features, possibly in different body parts. Consequently, differences between truthful and deceptive accounts are more often statistically significant when considering multiple body parts. The most discriminating features seem to be those that average over all body parts, such as the average movement or the average symmetry. This seems to suggest that global information is more reliable than local information, at least when measured over the entire duration of the interaction.

**Table 7:** Percentage of selected stat-95 features when training on both session types and average classification rate for different numbers of involved body parts on both session types, Game sessions only and Wallet sessions only, using windows of 2.5 min

Body parts involved in feature	Selected (%)	Classification rate		
		Both (%)	Game (%)	Wallet (%)
Single body part	27.89	57.57	59.90	55.24
Two body parts	49.76	56.98	57.80	56.17
All body parts	70.56	61.18	62.28	60.08

#### Influence of Window Type

We also used different ways of aggregating feature values over windows. From Table 3, it becomes clear that the minimum value of a feature is often not significantly different between truthful and deceptive accounts. This applies to both the Game and Wallet sessions. Especially for longer windows, the probability that values of movement are close to zero is rather high. As such, it is difficult to distinguish between truth and lie conditions. The classification rate of minimum features is also lower compared to the other window types. In contrast, more mean, maximum, and standard deviation features are selected, and they also appear more promising in the classification of truths and lies. Still, the features are complementary in terms of performance. Different window types might become relevant for different window sizes. For smaller windows, the maximum or standard deviation might be more meaningful as these reflect sudden movement better.

#### Top-Performing Individual Features

Aggregated results provide insight in the feature and window type and the body parts that are most informative. Additionally, we can investigate the performance of individual features. To this end, we remove the majority voting over features and ran our analyses for each feature individually. The top-20 best performing features with their classification rates are summarized in Table 8. The left arm is directly involved in 14 of these top 20 features, with different feature and window types. Additionally, six of the top-performing features consider the full or upper body, including the left arm. Apparently, the upper body and in particular the left arm provide cues to distinguish truthful from deceptive behavior. Given that the majority of the people is right-handed, we expect that, also in the data we analyzed, most of the gesturing is performed with the right hand. This could lead to a larger variance in features of the right hand. At the same time, differences in left arm use might be more stable, allowing for identification of more erratic or, conversely, more controlled, movement.

**Table 8:** Classification rates of the top-20 best performing individual stat-95 features using windows of 2.5 min. For body parts, H = head, B = body, LA = left arm, RA = right arm, LL = left leg, RL = right leg

Feature	Rate (%)	Type	Window	Body part
Full body symmetry	69.44	Symmetry	Maximum	
Left shoulder movement	67.22	Movement	Mean	
Right hand–left hand distance	66.67	Distance	Range	
Left elbow movement	66.11	Movement	Mean	
Left arm movement	66.11	Movement	Mean	
Left shoulder	66.11	Movement	Standard deviation	
Upper body movement	66.11	Movement	Standard deviation	
Full body joint distance	65.56	Distance	Maximum	
Right elbow–left hand distance	65.56	Distance	Range	
Full body movement	65.56	Movement	Standard deviation	
Left shoulder symmetry	65.56	Symmetry	Standard deviation	
Full body joint distance	65.00	Distance	Range	
Left elbow movement	65.00	Movement	Standard deviation	
Upper body movement	64.44	Movement	Mean	
Left shoulder symmetry	64.44	Symmetry	Maximum	
Right hand–left elbow distance	63.89	Distance	Range	
Left shoulder movement	63.89	Movement	Maximum	
Right hand–left elbow distance	63.89	Distance	Maximum	
Left shoulder movement	63.89	Movement	Range	
Left arm movement	63.89	Movement	Standard deviation	

Some of the individual features have classification rates higher than when *all* or *stat-95* features are used. The additional availability of lower-scoring features negatively affects the overall classification rate. This might be a sign of overfitting, the phenomenon that a predictive model does not generalize to out-of-sample data. Alternatively, this might just be the result of comparing a large number of features. We discuss this issue in the next section.

In particular, the maximum symmetry of the whole body is the best performing feature. This feature takes into account all body parts, and reveals some information about seating posture, potentially as a result of posture shifts. This information is also partly revealed in the maximum and range of the full body joint distance features. Joint distances typically change when the interviewee is more expressive, for example during gesturing or when moving the legs. A final full body feature in the top-20 is the standard deviation of the movement. Expressive motion of the whole body is, again, the main driver for this feature.

#### Influence of Amount of Noise

Figure 3 shows an approximately linearly decreasing classification rate for increasing noise factor *r*. The *stat-95* and *stat-99* classification continues to outperform a full set of features also with noise added. It is likely that a smaller set of features would be even more robust, in line with the findings discussed for the influence of window length. The robustness to noise is reasonable. Even with added noise with two times the standard deviation, the stat-95 and stat-99 features outperform humans. Especially for larger windows, the mean features are not affected much as the added noise has a mean of zero. This, again, points at the value of long-term stable differences between truthful and deceptive behavior.

### Data Mining for Deception Detection

This paper reports an experiment in mining bodily cues to deception. Based on a large set of features, obtained using motion capture equipment, we have derived simple statistical classifiers to distinguish between truthful and deceptive accounts. Overall classification with all features yielded a classification performance of 60.00%, compared to a baseline of 50.00% and human performance of 52.80%. The selection of features based on their statistical difference between the two conditions resulted in a smaller set with an improved classification rate of 65.00–65.56%. We found a higher classification performance for the Game sessions, most likely due a higher experienced cognitive load as a result of the reverse-order questioning. In line with Blandón-Gitlin et al. (2015) and Vrij et al. (2008), this suggests that this type of interviewing indeed is a good way of eliciting cues.

Our results have been obtained without modeling the correlations between features, to better understand how and where pose and motion differences between liars and truth-tellers occur. As such, the obtained classification performance is likely not to be optimal. Combining weak classifiers, such as in bagging (Breiman, 1996) is likely to exploit complementary information, while suppressing the effect of significant yet uninformative features.

From a series of analyses, we found that features in the upper body, especially the arms, were more often significantly different between the truth and lie conditions. The majority of the individually top performing features related to the left arm. However, features from other body parts proved to be almost as informative in terms of classification performance. Features of a single body part scored 5–8% lower compared to the combination of features of all body parts. We therefore believe that different body parts contain complementary information. A similar observation can be made for the different feature types (e.g., movement or joint distance) and window types (e.g., mean and maximum). Despite different ratios of selected features for each type, the classification performances are comparable but consistently lower than when using all features. We also evaluated the effect of noise and found that classification rates decrease linearly with an increase in the standard deviation of the noise.

The latter finding is important when moving from motion captured data to body measurements from vision-based processing. While the estimation of body joint positions in image space has reached a reasonable level of accuracy (e.g., Cao et al., 2019; Xu et al., 2023), significant challenges are to be overcome to record joint positions accurately in 3D (Pavllo et al., 2019). Still, we anticipate that the increasing sophistication of motion analysis algorithms will reduce the performance gap between motion capture and video-based measurement in the near future (Chen et al., 2020). Our observation that a modest amount of noise does not strongly deteriorate the detection rate is important for the practical development of vision-based deception systems. Moreover, by relying on vision-based pose estimation, we are able to analyze more recordings. This will eventually help to relate the findings of this paper to other interaction contexts.

#### Limitations

In this work, as in many deception studies (Levine, 2018a), we have used and assumed an equal chance of truth and deception in our sessions. When moving from a lab setting to the real world, the prior probability of encountering a deceptive account is typically much lower. As a consequence, the number of false positives, or type 1 errors, typically increases. Specifically, it means that relatively more honest people will be classified as being deceptive. Moreover, being to separate unbalanced class distributions of high-dimensional features require more training data (Duda et al., 2000). Currently, we are not aware of any data mining deception work that has been applied to real world data with a realistic class distribution.

In a practical application, an interaction is typically not entirely truthful or entirely deceptive. When analyzing the veracity of entire interactions, we cannot make distinctions at a finer timescale. While it is technically possible to provide veracity predictions at the level of a statement, it is likely that the performance is low, given that using smaller windows reduces the classification performance. Moreover, it remains an open problem how to train a classifier when veracity information is only available at the interaction level.

The main limitation of the current work in terms of the detection rate is the classification using majority voting of individually classified features. We have thus ignored potential correlations between these features. Moreover, we have not looked specifically at features with complementary information. We have found that feature type, place on the body, window length, and window type each carry partly complementary information that improves the classification. Exploiting combinations of features could yield increased and more stable classification rates. Also, we have considered features at a single temporal scale: we have not combined features across window sizes. It is likely that some discriminative movements are more salient in one temporal scale while other movements are more prominent in another. For shorter time windows, we will then face the issue that many window will not contain discriminative body movement. A more sophisticated way of training a classifier that decides for each training sample whether it is informative could alleviate this problem. In this case, only a small subset of the windows should contain discriminative information. Such an approach could be used to discover informative body movement patterns that happen irregularly. There is a risk that the flexibility of such a classification approach, in combination with a modest amount of training data, introduces overfitting: the increased probability of discovering incidental patterns. We stress the importance of a suitable mechanism to combat overfitting.

There might also be patterns of behaviors over time. These patterns can be mined automatically as well, and have been shown to be promising in distinguishing truthful from deceptive accounts (Burgoon et al., 2015; Duran et al., 2013). A combination of our work with the mining of patterns seems a fruitful way to discover discriminative patterns of behavior. In addition, the interviewer could be taken into account (Van der Zee et al., 2021). Especially when we take into account who is speaking when, the dynamics regarding turn-taking and the specific behaviors during listening and speaking could be analyzed. Moreover, we can analyze, and potentially account for, differences in interviewer behavior that might affect the interviewees’ behavior.

### Combining Data-Driven and Theory-Driven Research

While we have not correlated our findings to deception theories, there is clear merit for theory validation and developed using a data mining approach. In this paper, we have gained some insight into potential factors that explain discrepant findings between Duran et al. (2013) and Van der Zee et al. (2019). The length of the window—the amount of time that is considered—plays a crucial role in explaining detection rates. In Duran et al. (2013), the bodily behavior in short time windows has been explored and no differences were found between truth tellers and liars in the amount of movement. In this paper, we have observed that discrimination becomes more difficult when the time window decreases. Since Van der Zee et al. (2019) used windows of 2.5 min, more general patterns of aggregate movement have been taken into account. Moreover, we see that top informative features include full body features including the maximum and range of joint distance and the standard deviation of the movement. These suggest that both the amount of full body movement as well as the variation, seem to be relevant predictors for veracity. To fully understand how differences in data coding and experiment setup might have affected the detection rates, we would have to perform cross-dataset evaluation. This point has been raised as well by Levine (2018a), and is a more common research practice in data mining research. The major obstacle for cross-dataset evaluation is the lack of public data repositories for deception research; the availability of the data of Van der Zee et al. (2019) is an exception. While privacy of the recorded participants in the experiment is key, automatically recorded body pose data as used in this paper does not carry identifiable information. We therefore encourage researchers to make their coded behavior data available.

Correlating automatically—objectively—coded behavior to manually—subjectively—annotated data would further the analysis of discrepant findings. It could highlight whether differences between studies in terms of the coding scheme and coding conventions partly explain contrasting findings. Such insights help to improve manual coding practice. Conversely, when we understand how mined features relate to relevant discrete behaviors, we could make the mining process more context dependent. For example, we could take into account whether a person is talking, or seated.

## Conclusion

In this paper, we have presented a data mining approach to cues extracted from body movement data, with the aim of distinguishing truthful from deceptive behavior. In a systematic experimental study, we have investigated how detection rates are affected by considering different body parts, coding of the pose and movement, observation window lengths, and the amount of measurement noise. Using the simple classification of individual features, we have obtained detection rates above 65%, well outperforming human judgements (52.80%). Moreover, our systematic analyzes provide insight into the influence of various coding options on the deception detection rate. These results highlight the feasibility and merits of data-driven innovations in deception research. At the same time, there is ample room for improvements in terms of the classification performance.

Our study has further demonstrated that we can pinpoint and reconcile seemingly discrepant results across studies. Advances in the automated coding of 3D bodily behavior from camera footage will further facilitate the analysis and understanding of the bodily manifestations of deception. An increased availability of publicly available behavior data would finally allow cross-dataset evaluation, to ensure that findings generalize to across studies. We expect that such measures will allow researchers to quicker identify and resolve seemingly discrepant findings, to direct research to novel challenges, and to spark a renewed confidence in deception research.

## Data Availability

The datasets analyzed for this study can be found in the public Github repository of Van der Zee et al. (2019): https://github.com/sophievanderzee/To-freeze-or-not-to-freeze.

## References

[CR1] Avola D, Cinque L, De Marsico M, Fagioli A, Foresti GL (2020). LieToMe: Preliminary study on hand gestures for deception detection via Fisher-LSTM. Pattern Recognition Letters.

[CR2] Baltrušaitis T, Ahuja C, Morency L-P (2019). Multimodal machine learning: A survey and taxonomy. IEEE Transactions on Pattern Analysis and Machine Intelligence.

[CR3] Blandón-Gitlin I, Fenn E, Masip J, Yoo A (2015). Cognitive-load approaches to detect deception: Searching for cognitive mechanisms. Trends in Cognitive Sciences.

[CR4] Bond CF, DePaulo BM (2006). Accuracy of deception judgments. Personality and Social Psychology Review.

[CR5] Breiman L (1996). Bagging predictors. Machine Learning.

[CR6] Brennan T, Magnussen S (2020). Research on non-verbal signs of lies and deceit: A blind alley. Frontiers in Psychology.

[CR7] Buller DB, Burgoon JK (1996). Interpersonal deception theory. Communication Theory.

[CR8] Burgoon JK, Schuetzler R, Wilson DW (2015). Kinesic patterning in deceptive and truthful interactions. Journal of Nonverbal Behavior.

[CR9] Cao Z, Hidalgo Martinez G, Simon T, Wei S, Sheikh Y (2019). OpenPose: Realtime multi-person 2D pose estimation using part affinity fields. IEEE Transactions on Pattern Analysis and Machine Intelligence.

[CR10] Castellano, G., Villalba, S. D., & Camurri, A. (2007). Recognising human emotions from body movement and gesture dynamics. In A. C. R. Paiva, R. Prada, & R. W. Picard (Eds.), *ACII 2007: Affective Computing and Intelligent Interaction* (pp. 71–82). Springer. 10.1007/978-3-540-74889-2_7

[CR11] Chen Y, Tian Y, He M (2020). Monocular human pose estimation: A survey of deep learning-based methods. Computer Vision and Image Understanding.

[CR12] Constâncio AS, Tsunoda DF, Silva HFN, Silveira JM, Carvalho DR (2023). Deception detection with machine learning: A systematic review and statistical analysis. PLoS ONE.

[CR13] Dael N, Mortillaro M, Scherer KR (2012). The body action and posture coding system (BAP): Development and reliability. Journal of Nonverbal Behavior.

[CR14] Davis M, Markus K, Walters S, Vorus N, Connors B (2005). Behavioral cues to deception vs. topic incriminating potential in criminal confessions. Law and Human Behavior.

[CR15] de Turck MA, Miller GR (1985). Deception and arousal: Isolating the behavioral correlates of deception. Human Communication Research.

[CR16] Deeb H, Vrij A, Leal S, Fallon M, Mann S, Luther K, Granhag PA (2022). Sketching routes to elicit information and cues to deceit. Applied Cognitive Psychology.

[CR17] Denault V, Talwar V, Plusquellec P, Larivière V (2022). On deception and lying: An overview of over 100 years of social science research. Applied Cognitive Psychology.

[CR18] DePaulo BM, Lindsay JJ, Malone BE, Muhlenbruck L, Charlton K, Cooper H (2003). Cues to deception. Psychological Bulletin.

[CR19] Ding, M., Zhao, A., Lu, Z., Xiang, T., & Wen, J. R. (2019). Face-focused cross-stream network for deception detection in videos. In A. Gupta, D. Hoiem, G. Hua, & Z. Tu (Eds.), *Proceedings of the Conference on Computer Vision and Pattern Recognition* (pp. 7802–7811). IEEE Computer Society. 10.1109/CVPR.2019.00799

[CR20] Duda RO, Hart PE, Stork DG (2000). Pattern classification.

[CR21] Dunbar NE, Jensen ML, Tower DC, Burgoon JK (2014). Synchronization of nonverbal behaviors in detecting mediated and non-mediated deception. Journal of Nonverbal Behavior.

[CR23] Duran ND, Dale R, Kello CT, Street CNH, Richardson DC (2013). Exploring the movement dynamics of deception. Frontiers in Psychology.

[CR22] Duran ND, Fusaroli R (2017). Conversing with a devil’s advocate: Interpersonal coordination in deception and disagreement. PLoS ONE.

[CR24] Ekman P, Friesen WV (1969). Nonverbal leakage and clues to deception. Psychiatry.

[CR25] Ekman P, Friesen WV (1978). Facial action coding system: A technique for the measurement of facial movement.

[CR26] Elkins AC, Zafeiriou S, Pantic M, Burgoon JK, Calvo R, D’Mello S, Gratch J, Kappas A (2014). Unobtrusive deception detection. The Oxford handbook of affective computing.

[CR27] Hauch V, Sporer SL, Michael SW, Meissner CA (2016). Does training improve the detection of deception? A meta-analysis. Communication Research.

[CR28] Jensen ML, Meservy TO, Burgoon JK, Nunamaker JF, Chen H, Yang CC (2008). Video-based deception detection. Intelligence and security informatics: Techniques and applications.

[CR29] Kleinberg B, Verschuere B (2021). How humans impair automated deception detection performance. Acta Psychologica.

[CR30] Kleinsmith A, Bianchi-Berthouze N (2013). Affective body expression perception and recognition: A survey. IEEE Transactions on Affective Computing.

[CR31] Levine T (2018). Ecological validity and deception detection research design. Communication Methods and Measures.

[CR32] Levine T (2018). Scientific evidence and cue theories in deception research: Reconciling findings from meta-analyses and primary experiments. International Journal of Communication.

[CR33] Levine T, Feeley T, McCornack S, Hughes M, Harms C (2005). Testing the effects of nonverbal behavior training on accuracy in deception detection with the inclusion of a bogus control group. Western Journal of Communication.

[CR34] Lu, S., Tsechpenakis, G., Metaxas, D. N., Jensen, M. L., & Kruse, J. (2005). Blob analysis of the head and hands: A method for deception detection. In J. F. Nunamaker Jr., & R. O. Briggs (Eds.), *Proceedings of the Hawaii International Conference on System Sciences*. IEEE Computer Society. 10.1109/HICSS.2005.122

[CR35] Luke TJ (2019). Lessons from Pinocchio: Cues to deception may be highly exaggerated. Perspectives on Psychological Science.

[CR36] Meissner C, Kassin S (2002). “He’s guilty!”: Investigator bias in judgments of truth and deception. Law and Human Behavior.

[CR37] Pavllo, D., Feichtenhofer, C., Grangier, D., & Auli, M. (2019). 3D human pose estimation in video with temporal convolutions and semi-supervised training. In A. Gupta, D. Hoiem, G. Hua, & Z. Tu (Eds.), *Proceedings of the Conference on Computer Vision and Pattern Recognition* (pp. 7753–7762). IEEE Computer Society. 10.1109/CVPR.2019.00794

[CR38] Poppe R, Burgoon JK, Magnenat-Thalmann N, Pantic M, Vinciarelli A (2017). Automatic analysis of bodily social signals. Social signal processing.

[CR39] Poppe R, Van der Zee S, Heylen DKJ, Taylor PJ (2014). AMAB: Automated measurement and analysis of body motion. Behavior Research Methods.

[CR40] Serras Pereira M, Cozijn R, Postma E, Shahid S, Swerts M (2016). Comparing a perceptual and an automated vision-based method for lie detection in younger children. Frontiers in Psychology.

[CR41] Sporer S, Schwandt B (2007). Moderators of nonverbal indicators of deception: A meta-analytic synthesis. Psychology, Public Policy, and Law.

[CR42] Sánchez-Monedero J, Dencik L (2022). The politics of deceptive borders: ‘Biomarkers of deceit’ and the case of iBorderCtrl. Information, Communication & Society.

[CR43] Van der Zee S, Poppe R, Taylor PJ, Anderson RJ (2019). To freeze or not to freeze: A culture-sensitive motion capture approach to detecting deceit. PLoS ONE.

[CR44] Van der Zee S, Taylor P, Wong R, Dixon J, Menacere T (2021). A liar and a copycat: Nonverbal coordination increases with lie difficulty. Royal Society Open Science.

[CR45] Vrij A (2008). Detecting lies and deceit: Pitfalls and opportunities.

[CR46] Vrij A, Granhag PA (2012). Eliciting cues to deception and truth: What matters are the questions asked. Journal of Applied Research in Memory and Cognition.

[CR47] Vrij A, Granhag PA, Porter S (2010). Pitfalls and opportunities in nonverbal and verbal lie detection. Psychological Science in the Public Interest.

[CR48] Vrij A, Hartwig M, Granhag PA (2019). Reading lies: Nonverbal communication and deception. Annual Review of Psychology.

[CR49] Vrij A, Mann S, Fisher R, Leal L, Milne B, Bull R (2008). Increasing cognitive load to facilitate lie detection: The benefit of recalling an event in reverse order. Law and Human Behavior.

[CR50] Wu, Z., Singh, B., Davis, L., & Subrahmanian, V. (2018). Deception detection in videos. In S. A. McIlraith, & K. Q. Weinberger (Eds.), *Proceedings of the AAAI Conference on Artificial Intelligence* (pp. 1695–1702). AAAI Press. 10.5555/3504035.3504242

[CR51] Xu L, Jin S, Liu W, Qian C, Ouyang W, Luo P, Wang X (2023). ZoomNAS: Searching for whole-body human pose estimation in the wild. IEEE Transactions on Pattern Analysis and Machine Intelligence.

[CR52] Yarkoni T, Westfall J (2017). Choosing prediction over explanation in psychology: Lessons from machine learning. Perspectives on Psychological Science.

[CR53] Zhou L, Burgoon JK, Twitchell DP, Qin T, Nunamaker JF (2004). A comparison of classification methods for predicting deception in computer-mediated communication. Journal of Management Information Systems.

